# Integrative Approach to Somatic (Psychogenic) Cough in a Geriatric Female Patient: A Case Study

**DOI:** 10.7759/cureus.95760

**Published:** 2025-10-30

**Authors:** Payam Bokhoor

**Affiliations:** 1 Internal Medicine, UCLA Center for East-West Medicine, Los Angeles, USA

**Keywords:** acupuncture, chronic cough, integrative medicine, lung qi deficiency, somatic/psychogenic cough syndrome

## Abstract

Chronic cough, defined as a cough lasting more than eight weeks, is a common condition that can significantly impair one’s quality of life. Chronic cough is often attributed to respiratory, gastrointestinal, or allergic causes; however, a subset of patients experience persistent symptoms without identifiable pathology. In such cases, somatic cough syndrome (formerly known as psychogenic cough) should be considered.

This report describes the successful treatment of a chronic refractory cough in an elderly patient using an integrative East-West medical approach. Following assessments by multiple specialists and a negative workup, along with a clinical history marked by unresolved grief, a diagnosis of somatic (psychogenic) cough was established. Non-pharmacologic therapies including acupuncture, dietary changes, insight-based guidance, and mind-body practices led to substantial symptom improvement. Clinicians should consider somatic (psychogenic) cough in cases of chronic cough that remain unresponsive to standard therapies. Integrative medicine offers a patient‑centered, holistic approach to managing complex and multifactorial cases.

## Introduction

Chronic cough is a common yet complex condition that can significantly impact a patient’s quality of life. It is defined as a cough lasting more than eight weeks and is commonly associated with conditions such as chronic respiratory infections, asthma, gastroesophageal reflux disease, chronic obstructive pulmonary disease (COPD), medications, and post-nasal drip [1-3]. However, a subset of patients experience persistent cough without an identifiable organic cause, despite a comprehensive workup. This subgroup may be classified as having psychogenic cough or, as more recently termed in the DSM-5, somatic cough syndrome. Somatic cough is a form of chronic cough that is driven by psychological factors such as stress, anxiety, or emotional distress and often poses a therapeutic challenge [4].

Integrative medical approaches that combine conventional and complementary therapies may provide relief, particularly in patients with an emotional or psychological component to their symptoms. This case report discusses the presentation, workup, and management of an elderly woman with a refractory chronic cough who experienced significant improvement of her symptoms through an integrative East-West Medicine approach.

## Case presentation

**Figure 1:**
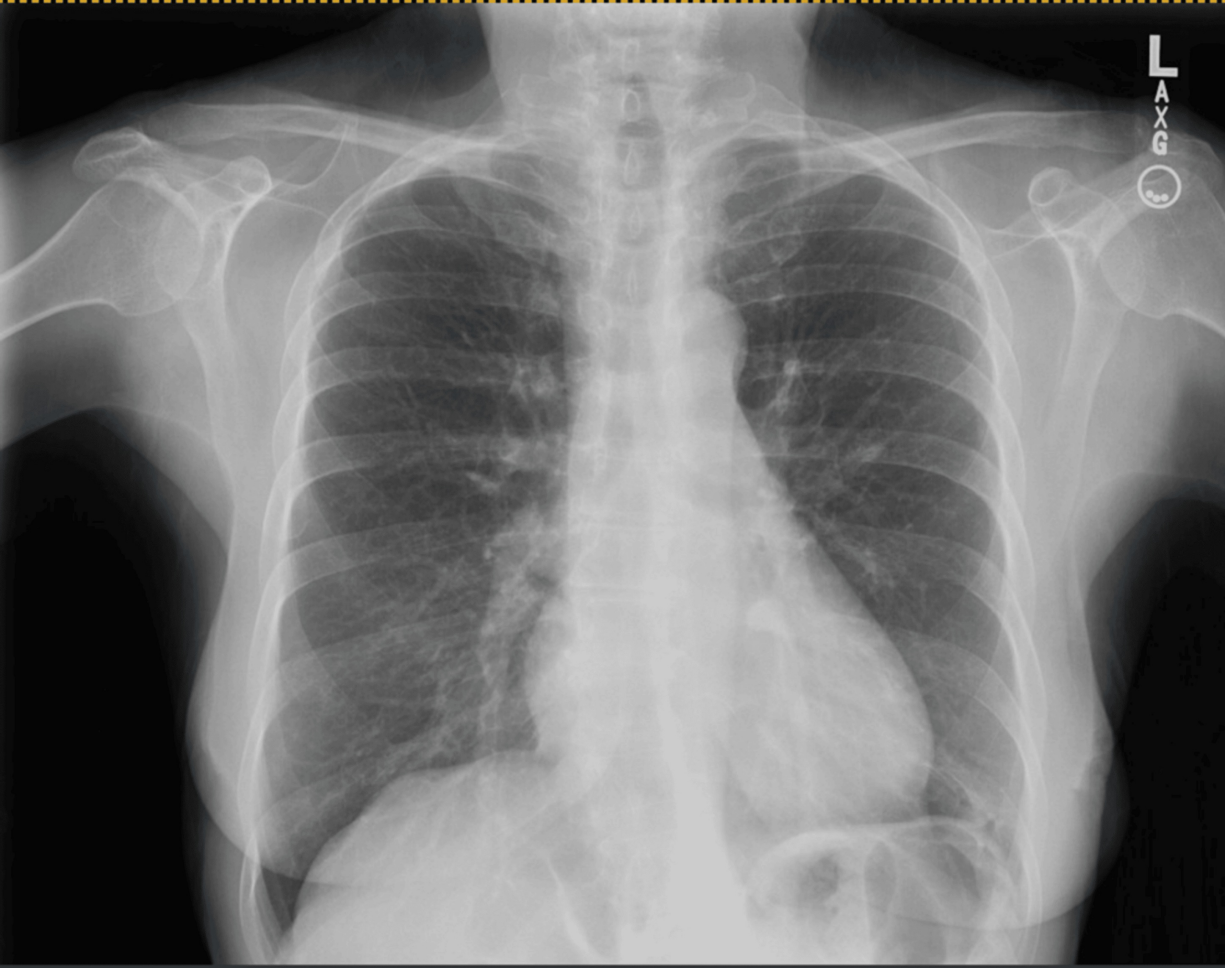
A chest X-ray without any significant findings to explain chronic cough.

A 72-year-old woman with a medical history of hypertension and osteoarthritis presented to the outpatient clinic with a chronic, non-productive cough persisting for over six months. The cough was not associated with hemoptysis or dyspnea. She denied any significant weight loss, fever, or night sweats. There was no history of tobacco use, environmental exposure to irritants, or recent travel. Her only medication was amlodipine 5mg and Tylenol as needed for pain. The patient reported that the cough occurred intermittently throughout the day, worsening in the morning and during periods of emotional stress. She experienced frequent mild coughing spells during prolonged conversations and social interactions, which led to embarrassment and social withdrawal. A thorough diagnostic workup including pulmonary function tests, chest X-ray (Figure 1), and chest CT revealed no significant evidence of COPD, interstitial lung disease, or malignancy.

She was placed on a short-term trial of inhaled corticosteroids, nasal steroids, and nasal irrigation by a pulmonologist without improvement. She was then referred to a gastroenterologist and underwent a trial of acid-suppressive medications and dietary modifications, which also provided no benefit. An esophagogastroduodenoscopy (EGD) and 24-hour pH monitoring were performed, both yielding negative results for gastrointestinal pathology. Finally, allergy testing was conducted and was negative for any allergens that could explain her symptoms.

Given the refractory nature of her symptoms and negative workup, she was referred to the UCLA Center for East-West Medicine for alternative treatment strategies. During the initial assessment, a comprehensive integrative biopsychosocial approach was undertaken. The patient reported significant emotional stress and ongoing grief following the death of her spouse the previous year. She described a profound connection with her late husband and a persistent sense of emptiness since his passing. Further questioning revealed the cough developed several months after his passing, with no other identifiable inciting triggers. In-clinic surveys were negative for depression and generalized anxiety disorder. Based on the extensive negative workup and her current presentation, she was diagnosed with psychogenic (somatic) cough.

Initially, a trial of cognitive-behavioral therapy was recommended but discontinued because she found it triggering and emotionally distressing. Additionally, she wished to avoid pharmacologic and psychiatric medications. A Traditional Chinese Medicine (TCM) framework was then employed, which helped explain her symptoms in simpler terms as a Lung Qi deficiency, which is a condition that can be triggered by significant grief or sadness, resulting in emotional blockage and Qi stagnation. Acupuncture was initially administered twice a month to support her emotional well-being and to work on the lung meridian, aiming to improve Qi stagnation. She was given TCM dietary recommendations to reduce dairy products and greasy foods while increasing her intake of whole grains, nuts, cinnamon, fennel, steamed Asian pears, ginseng, and chrysanthemum tea to help tonify the Lung Qi deficiency. Mindfulness and breathing exercises were also recommended in the form of qigong, lung meridian tapping, and acupressure. After eight weeks, she reported approximately a 40% improvement in her cough. At that time, acupuncture treatments were reduced to once monthly, and she was encouraged to gradually re-engage in social circles and activities that she previously avoided due to embarrassment caused by the cough.

At six months, she reported only rare episodes of coughing that no longer interfered with her conversations or social interactions. She was no longer distressed by the cough and was able to effectively apply the coping strategies she had learned earlier to reduce exacerbations during times of stress. Overall, she described feeling “renewed and extremely grateful, with a 90% improvement in my cough.”

## Discussion

Chronic cough, defined as a cough lasting more than eight weeks, affects an estimated 10-12% of the adult population globally, with higher prevalence reported among women and older adults [5,6]. The symptom is not only physically taxing but also socially and emotionally disruptive. Many patients report embarrassment, sleep disturbances, fatigue, and significant impairment in quality of life [7]. In fact, chronic cough has been associated with depression, anxiety, and social isolation, especially when a definitive cause cannot be identified and symptoms are refractory to treatment [8,9].

The diagnosis of psychogenic cough can be considered in patients like the one presented. Psychogenic cough, also referred to as somatic cough syndrome in DSM-5, typically emerges in the context of emotional distress and lacks an identifiable organic pathology [4,10]. It is often characterized by a dry, repetitive cough that improves during sleep or with distraction. In contrast, habit cough is more commonly diagnosed in children and is thought to be a learned behavior that may respond well to behavioral therapy techniques such as suggestion therapy [10].

TCM provides a meaningful framework for understanding and treating her condition. This patient’s cough onset coincided with the loss of her spouse and was compounded by ongoing grief and emotional suppression. TCM attributes chronic cough to a dysfunction in lung Qi, often exacerbated by emotional imbalances such as grief, which is known to "injure" the lung in TCM theory [11-12]. Chronic grief is thought to weaken lung Qi, leading to persistent cough and fatigue [11]. To help address her symptoms, dietary recommendations focused on nourishing and tonifying lung Qi while addressing emotional imbalances. Foods such as steamed Asian pears, fennel seeds, and American ginseng were incorporated for their moistening and Qi-tonifying properties. Dairy intake was reduced, as it is believed in TCM to contribute to dampness and phlegm accumulation that can exacerbate lung dysfunction [13]. Chrysanthemum tea, traditionally used to clear heat and calm the liver, was also recommended for its calming effects and potential to alleviate emotional tension [14].

Furthermore, acupuncture has demonstrated effectiveness in treating chronic cough and emotional dysregulation [15]. Studies suggest that acupuncture modulates the autonomic nervous system, reduces inflammatory mediators, and improves airway function. It has also been shown to influence brain regions related to emotional regulation, supporting its role in psychosomatic conditions [15-16]. Qigong is a meditative movement practice that integrates breath control and body awareness, supporting both emotional and respiratory regulation. Previous studies have demonstrated qigong’s benefits in reducing stress and improving symptoms in chronic illnesses [17].

As the patient improved, a key component of the integrative approach was encouraging her to gradually re-engage in meaningful social interactions and community activities, thereby addressing the emotional isolation and loss of identity she experienced following the death of her husband. Reconnection with supportive networks has been shown to improve emotional resilience and may play a therapeutic role in reducing psychogenic symptoms, particularly in older adults coping with grief [18].

## Conclusions

This case highlights the complexity of diagnosing and treating chronic cough, especially when no clear etiology is identifiable. Despite the absence of objective findings through standard testing, the patient’s chronic cough was ultimately diagnosed as a psychogenic (somatic) cough, likely exacerbated by underlying stress and grief. In cases where conventional diagnostic evaluations are inconclusive, clinicians should consider the possibility of a psychogenic (somatic) cough, particularly when there is evidence of psychological distress.

This outcome demonstrates the value of an integrative medical approach. Integrative therapies emphasize patient-centered care and recognize the interconnection between emotional, physical, and social dimensions of health. In this instance, validating the emotional roots of the patient’s condition and addressing them through both Eastern and Western paradigms led to significant symptom improvement. This illustrates how a holistic approach can achieve favorable outcomes, particularly in patients who are refractory to conventional therapies.
